# Pulmonary tularemia presenting as bilateral pulmonary nodules during BEP chemotherapy for testicular embryonal carcinoma: A diagnostic challenge

**DOI:** 10.1016/j.radcr.2026.06.012

**Published:** 2026-06-29

**Authors:** Ghazal Shadmani

**Affiliations:** Washington University School of Medicine, Mallinckrodt Institute of Radiology, 660 S. Euclid Ave., MSC 8131-0050-05, St. Louis, MO 63110, USA

**Keywords:** Pulmonary tularemia, *Francisella tularensis*, Testicular cancer, Zoonotic infection, PET-CT, Bleomycin induced pulmonary toxicity

## Abstract

Pulmonary tularemia is a rare zoonotic infection caused by *Francisella tularensis* that can mimic malignancy on imaging. This report presents a 38-year-old man with testicular embryonal carcinoma who developed multiple bilateral pulmonary nodules approximately 2 and a half weeks after initiating bleomycin, etoposide, and cisplatin (BEP) chemotherapy. The imaging differential diagnoses included pulmonary metastatic disease, bleomycin-induced pulmonary toxicity (BIP), and infection. Although the aggressive biology of embryonal carcinoma made pulmonary metastasis a significant concern, the poorly marginated nodule morphology and peribronchovascular distribution were atypical for hematogenous metastasis, and normalized serum tumor markers argued against active metastatic disease. Positive *F. tularensis* serology combined with zoonotic exposure history ultimately established the diagnosis of pulmonary tularemia. The patient had epidemiological exposure through outdoor dogs that hunted small animals. Following treatment with oral ciprofloxacin for 10 days, follow-up CT at 1 month demonstrated significant improvement with decreased size and number of pulmonary nodules and pleural effusions. This case highlights the importance of recognizing atypical nodule morphology, correlating imaging findings with tumor markers, and considering the timing of nodule appearance relative to chemotherapy to avoid misdiagnosis, inappropriate staging, and unnecessary treatment escalation in oncology patients.

## Introduction

Tularemia is a zoonotic infection caused by *Francisella tularensis*, a highly virulent gram-negative coccobacillus that affects humans and small wild animals [1]. The disease is endemic throughout the Northern Hemisphere, with the highest incidence in the United States occurring in south-central and western states, particularly Missouri, Arkansas, Oklahoma, South Dakota, and Montana [2]. Human infection occurs through arthropod bites, handling infected animal tissues, direct environmental exposure, or inhalation of infective aerosols [1,3]. Pulmonary tularemia is a rare but potentially severe manifestation that may result from inhalational exposure or hematogenous spread from a distant site [3]. Reported imaging findings include patchy airspace opacities, hilar or mediastinal lymphadenopathy, pleural effusion, and less commonly cavitary or miliary pulmonary disease resembling tuberculosis [3,4]. More recent CT studies have additionally described pulmonary nodules, sometimes associated with subpleural micronodules or pleural effusion [4,5]. However, pulmonary nodules as the dominant radiographic manifestation remain uncommon and have been described in only a limited number of reports, many of which lack detailed imaging characterization [6]. Pulmonary tularemia may closely mimic thoracic malignancy, particularly on [^18^F]-fluorodeoxyglucose (FDG) positron emission tomography (PET) FDG PET/CT, where hypermetabolic pulmonary lesions can be indistinguishable from primary or metastatic neoplastic disease. CT findings have been interpreted as suspicious for malignancy in up to 77% of patients with pulmonary tularemia [7]. This diagnostic challenge is especially important in patients with known malignancy, in whom newly detected pulmonary nodules are frequently presumed to represent metastatic disease, potentially resulting in inaccurate staging, unnecessary treatment escalation, or delay in appropriate antimicrobial therapy. In patients undergoing chemotherapy, the differential diagnosis may be further confounded by treatment-related pulmonary toxicity, including BIP and opportunistic infections [8]. Herein, an unusual case of pulmonary tularemia presenting as bilateral pulmonary nodules in a patient with testicular embryonal carcinoma undergoing chemotherapy is presented. By providing detailed serial CT imaging documenting the morphologic appearance, temporal evolution, and treatment response of the pulmonary nodules, this report highlights imaging and clinical clues that may assist radiologists and oncologists in distinguishing this treatable infection from metastatic disease and avoiding unnecessary oncologic interventions.

## Case report

A 38-year-old man diagnosed with testicular embryonal carcinoma via right testicular mass biopsy. His medical history was significant for chronic hepatitis C virus (HCV) infection, prior incarceration, and history of intravenous drug use. Importantly, the patient owned 2 dogs that were primarily kept outdoors and frequently hunted small animals including rabbits and rodents. Preoperative serum tumor markers showed elevated alpha-fetoprotein (AFP) and beta-human chorionic gonadotropin (β-hCG). A baseline chest CT showed an indeterminate right lower lobe 3 mm nodule. He underwent radical orchiectomy with pathology confirming embryonal carcinoma.

**Fig. 1:**
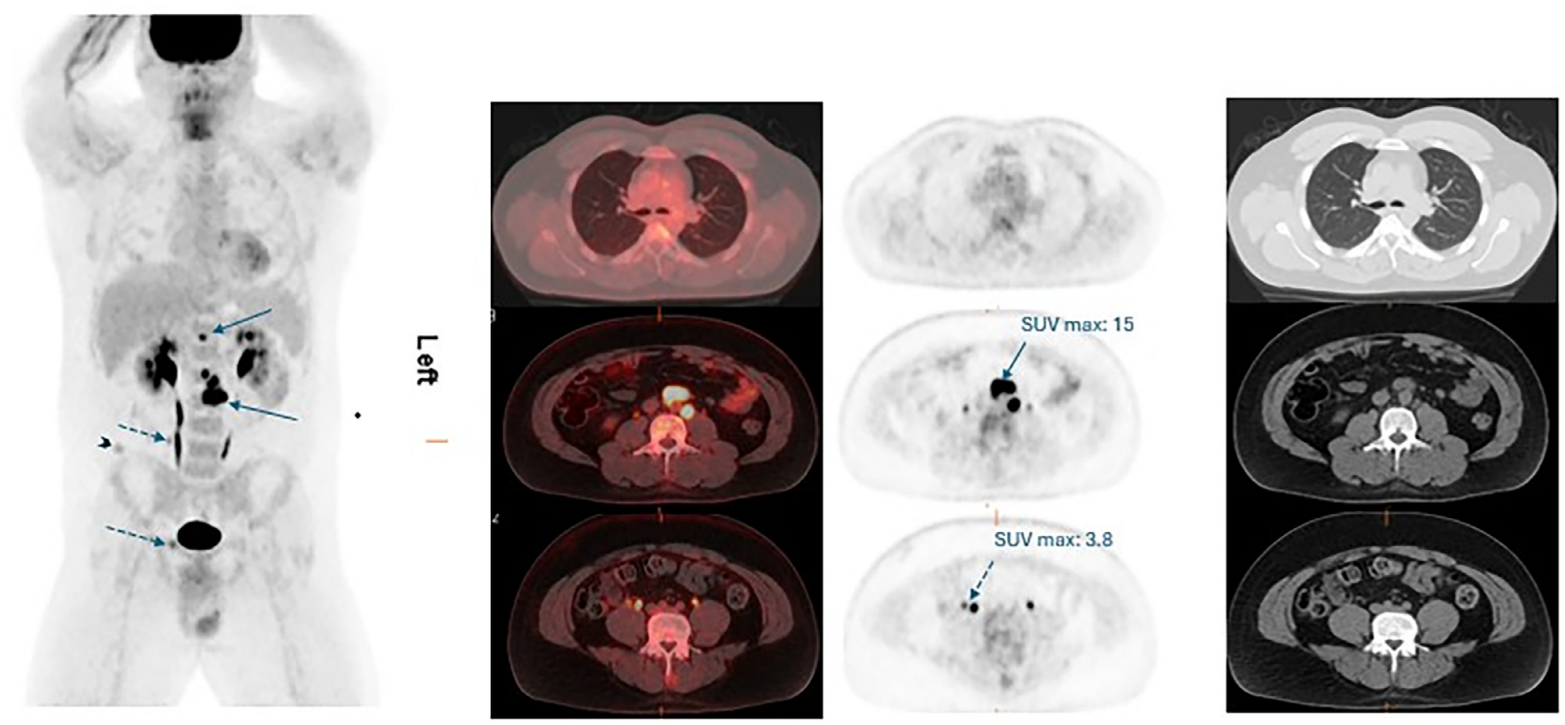
Coronal maximum intensity projection (MIP) FDG PET image obtained for staging following radical orchiectomy for testicular embryonal carcinoma. Markedly hypermetabolic retroperitoneal lymph nodes (SUV max: 15) consistent with nodal metastatic disease are identified (solid arrows). Moderately hypermetabolic right common iliac and right external iliac lymph nodes (dashed arrows) were considered indeterminate, with differential considerations including reactive/inflammatory changes versus nodal metastasis. Importantly, no suspicious FDG uptake is identified in the chest, and the lungs are clear without evidence of pulmonary metastatic disease at this time. Focal moderate FDG uptake projecting above the right iliac crest corresponds to a benign cutaneous inflammatory uptake (blue arrowhead), The remainder of the body shows no evidence of distant metastatic disease. Physiologic radiotracer activity is noted in the brain, myocardium, kidneys, and urinary bladder.

**Fig. 2:**
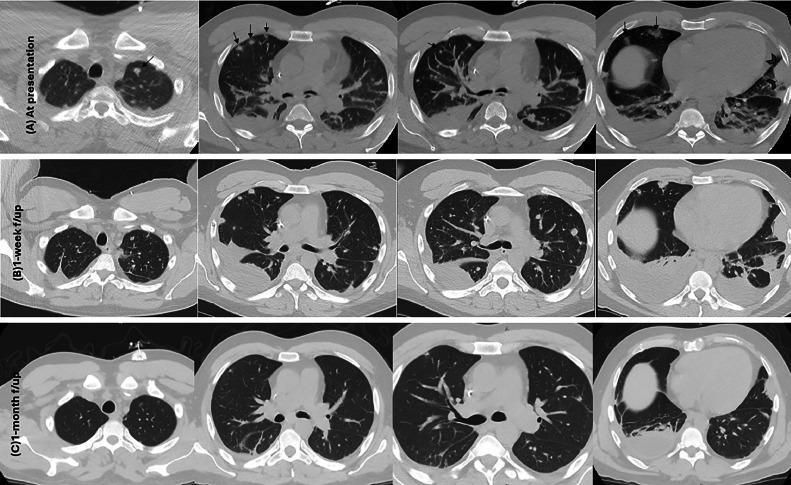
Axial CT images of the chest demonstrate temporal evolution of pulmonary findings. (A) At presentation of respiratory symptoms, multiple poorly defined (ill-marginated) bilateral pulmonary nodules, some with surrounding ground-glass opacity (arrows), lingula consolidative mass (arrow heads) with small bilateral pleural effusions. (B) One-week follow-up CT demonstrates persistent/ slightly increased bilateral pleural effusions with persistent pulmonary opacities. (C) One-month follow-up CT shows marked improvement of the pulmonary nodules, with residual pleural effusions.

FDG PET/CT was performed 1 week after orchiectomy (approximately 2 weeks after the baseline chest CT) for staging (Fig. 1). The previously noted indeterminate 3 mm right lower lobe nodule on the baseline chest CT was not well visualized on the low-dose CT component of the FDG PET/CT. The study demonstrated intensely hypermetabolic retroperitoneal (aortocaval and pre/para-aortic) and right retrocrural lymph nodes consistent with nodal metastatic disease. Moderately hypermetabolic right common iliac and right external iliac lymph nodes were also noted, with differential considerations including reactive or post-surgical changes versus nodal metastasis. No suspicious FDG uptake was identified in the chest, and the lungs were clear without evidence of pulmonary metastatic disease. No pelvic lymph node involvement was identified. Subsequently, the patient was initiated on BEP chemotherapy. Approximately 2 and a half weeks after the first dose of chemotherapy, the patient presented to the emergency department with tachycardia, nausea/vomiting, and acute kidney injury. Chest CT (Fig. 2A) revealed numerous bilateral new pulmonary nodules. The nodules ranged from 5 to 15 mm in diameter, demonstrated poorly marginated, ill-defined borders with a peribronchovascular distribution throughout both lungs and no zonal predominance. While the 2-month interval from the staging PET/CT was sufficient for the potential development of new metastatic disease, particularly given the aggressive biology of embryonal carcinoma, the close temporal relationship between chemotherapy initiation and the onset of respiratory symptoms and new pulmonary findings also raised significant concern for either BIP toxicity or an infectious process. Bilateral pleural effusions were also present. Follow-up chest CT performed 1 week later (Fig. 2B) demonstrated stable size of the numerous pulmonary nodules with increased bilateral pleural effusions. There was also questionable minimal cavitation of 1 nodule. Serum tumor markers were checked which were not elevated. This finding argued strongly against active metastatic disease, as serum tumor markers are highly sensitive indicators of disease activity in nonseminomatous germ cell tumors [9]. The discordance between the appearance of new pulmonary nodules and normalized tumor markers was a critical clinical finding that prompted further investigation for alternative diagnoses. Bronchoalveolar lavage was positive for respiratory syncytial virus (RSV), though contamination was considered possible. Acid-fast bacilli smears and cultures were negative. Tuberculosis PCR was negative. Fungal studies were negative. *F. tularensis* serology showed positive IgG and IgM antibodies. The positive tularemia serology, combined with the patient’s exposure history of owning outdoor dogs that hunted small animals, raised strong suspicion for pulmonary tularemia. Serology is the most common method for confirming tularemia diagnosis [7,10]. The patient resided in Missouri, one of the highest-incidence states for tularemia in the United States [2]. CT-guided lung biopsy was performed targeting one of the larger pulmonary nodules. Histopathologic examination revealed small areas of nodular fibrosis with mildly increased alveolar macrophages and no evidence of malignancy. Based on the positive tularemia serology and epidemiological exposure, the patient was treated with oral ciprofloxacin for 10 days. Bleomycin was permanently discontinued from the chemotherapy regimen given the initial concern for bleomycin-induced pulmonary toxicity.

Serial chest CT imaging documented the temporal evolution of the pulmonary findings. Follow-up chest CT obtained 2 weeks later showed slight decrease in size of the pulmonary nodules, and ciprofloxacin therapy was initiated at this time. Follow-up chest CT approximately 1 month after the initial presentation and 2 weeks after starting ciprofloxacin (Fig. 2C) demonstrated markedly decreased size of bilateral pulmonary nodules with resolution of the left pleural effusion; however, the right pleural effusion persisted, associated with focal pleural thickening and round atelectasis. Subsequent follow-up CT at approximately 2 and a half months after the initial presentation confirmed complete resolution of the right pleural effusion.

## Discussion

This case illustrates the diagnostic challenge of pulmonary tularemia presenting in a patient with known malignancy undergoing chemotherapy, where newly detected pulmonary nodules must be differentiated from metastatic disease, drug-induced pulmonary toxicity, and opportunistic infection. Several imaging, clinical, and temporal features were critical in raising suspicion for final diagnosis and ultimately establishing the correct diagnosis.

### Metastatic pattern and tumor biology

Testicular germ cell tumors demonstrate a predictable pattern of lymphatic dissemination, with regional metastases initially involving retroperitoneal lymph nodes below the renal vessels along the course of the testicular veins. Right testicular tumors typically spread to the paracaval, precaval, and aortocaval nodal stations, whereas left testicular tumors most commonly involve the left para-aortic nodes. Pelvic nodal metastases are uncommon in the absence of retroperitoneal lymphadenopathy [11,12].

Embryonal carcinoma is among the most aggressive nonseminomatous germ cell tumor subtypes, with a strong propensity for early hematogenous dissemination. The lungs represent the most common site of visceral metastasis secondary to hematogenous dissemination [9]. Therefore, the clinical urgency to rule out pulmonary metastasis in this patient was high.

### Imaging features distinguishing infection from metastases

The nodule morphology provided the key imaging clue. The poorly marginated, ill-defined borders and peribronchovascular distribution observed in this case were atypical for hematogenous metastases, which characteristically present as multiple, well-circumscribed, peripheral nodules with a random distribution unrelated to the airway [13]. A peribronchovascular nodular pattern is more characteristic of infectious or inflammatory processes that spread along the bronchovascular bundles. Few nodules demonstrated surrounding ground-glass opacity, a feature more suggestive of hemorrhagic or inflammatory process than metastatic deposits. Additionally, bilateral pleural effusions were present, which occur in approximately 30% of pulmonary tularemia cases [4]. Notably, mediastinal or hilar lymphadenopathy was absent in this case, which is atypical for pulmonary tularemia; recent case series have consistently reported mediastinal lymphadenopathy and pulmonary nodules as the most common CT findings [5].

### Bleomycin-induced pulmonary toxicity as a differential consideration

An important differential consideration was bleomycin-induced pulmonary toxicity (BIP), given the temporal relationship between chemotherapy initiation and the onset of respiratory symptoms. BIP most commonly manifests on CT as diffuse or multifocal ground-glass opacities, interlobular septal thickening, reticular changes, and areas of consolidation, often with a lower-lobe and subpleural predominance [8]. The incidence of pulmonary toxicity is proportionate to the cumulative bleomycin dose, occurring in approximately 10% of treated patients with fatal pulmonary fibrosis in approximately 1% [14]. Although bleomycin-induced pulmonary toxicity (BIP) most commonly manifests with interstitial and airspace opacities, a nodular variant has also been described, characterized by discrete pulmonary nodules that frequently demonstrate bronchiolitis obliterans organizing pneumonia (BOOP)/organizing pneumonia Histopathologically [15]. This nodular variant can closely mimic metastatic disease and represents a diagnostic pitfall. However, in the present case, the rapid radiographic resolution following antibiotic therapy with ciprofloxacin strongly favored an infectious etiology over bleomycin toxicity, as BIP would not be expected to respond to antimicrobial therapy. Bleomycin was nonetheless permanently discontinued from the chemotherapy regimen as a precautionary measure.

### Role of chemotherapy-induced immunosuppression

The temporal relationship between the infectious presentation and initiation of BEP chemotherapy raises the possibility that chemotherapy-induced immunosuppression contributed to the clinical manifestation of tularemia. BEP chemotherapy is associated with clinically significant myelosuppression, with reported febrile neutropenia rates ranging from approximately 5%-25% in patients receiving 3-4 cycles of therapy [13]. The patient presumably had prior subclinical or latent exposure to *F. tularensis* through his outdoor dogs that hunted small animals, and the chemotherapy-induced immunosuppression may have facilitated reactivation or clinical manifestation of the infection at this time.

### Limitations of histopathologic diagnosis

The absence of characteristic necrotizing granulomas or caseation necrosis may have been related to the patient’s immunocompromised state following initiation of BEP chemotherapy, which could have altered the expected inflammatory response. Alternatively, the biopsy may have been performed during an early stage or rapidly evolving phase of pulmonary tularemia before development of fully formed granulomatous changes. In this setting, the diagnosis was ultimately supported by the combination of positive *F. tularensis* serology, epidemiologic exposure history, exclusion of alternative infectious etiologies, and subsequent radiographic response to ciprofloxacin therapy.

## Conclusion

Pulmonary tularemia can closely mimic metastatic disease and bleomycin-induced pulmonary toxicity in patients undergoing chemotherapy. In this case, atypical nodule morphology, discordance with serum tumor markers, zoonotic exposure history, and rapid response to antibiotic therapy were critical in establishing the correct diagnosis. Awareness of this rare but treatable entity may help avoid inaccurate cancer staging, unnecessary treatment escalation, and delay in appropriate antimicrobial therapy.

## Patient consent

Written informed consent was obtained from the patient for publication of this case report and accompanying images.
